# Massive acetaminophen ingestion managed successfully with N-acetylcysteine, fomepizole, and renal replacement therapy 

**DOI:** 10.5414/CNCS111275

**Published:** 2024-03-02

**Authors:** Elizabeth E. Williams, Duc Quach, Arthur Daigh

**Affiliations:** Indiana University School of Medicine, Indianapolis, IN, USA

**Keywords:** acetaminophen, N-acetylcysteine, fomepizole, dialysis

## Abstract

Acetaminophen ingestion is routinely managed with the antidote, N-acetylcysteine (NAC). Massive acetaminophen poisoning has been treated successfully with adjunctive therapies such as fomepizole and hemodialysis. Fomepizole functions by inhibiting cytochrome p560, which prevents tylenol from forming its toxic metabolite, NAPQI. Prior cases have demonstrated favorable outcomes and a significant drop in acetaminophen levels after a single session of intermittent hemodialysis and continuous veno-venous hemofiltration (CVVH). However, the recommended dosage adjustments of NAC and fomepizole while a patient is undergoing CVVH has not been well reported. We present a case of an 18-year-old male who presented after ingesting 125 g of tylenol. His 4-hour acetaminophen level was 738.6 µg/mL. He was treated with NAC, fomepizole, and a single 4-hour session of hemodialysis. His acetaminophen level remained elevated at 730 µg/mL despite the hemodialysis session. CVVH was initiated, and he was given intravenous NAC at 12.5 mg/kg/h, oral NAC at 70 mg/kg every 4 hours, and intravenous fomepizole at 10 mg/kg every 6 hours. His tylenol levels became undetectable 57 hours after ingestion, and he did not develop permanent liver toxicity. This case encourages the use of CVVH for massive tylenol ingestion when a single run of intermittent hemodialysis is not effective in lowering the tylenol level. NAC, fomepizole, and CVVH can prevent unfavorable outcomes in massive acetaminophen ingestion when provided at an appropriate dose and frequency.

## Introduction 

Tylenol toxicity is the most common indication for liver transplantation in the United States and often develops with consumption of at least 7.5 – 10 g of acetaminophen (APAP) per day [1]. Massive acetaminophen poisoning is defined by an ingestion of at least 50 grams of APAP or an APAP level above 300 µg/mL at least 4 hours after ingestion [2]. Acetaminophen’s metabolite, NAPQI, is a hepatic toxin. It injures mitochondrial proteins within the liver, impairing the synthesis of ATP, which leads to hepatocellular necrosis and ultimately liver failure [3]. N-acetylcysteine (NAC) is the first-line treatment for APAP ingestion and works by restoring hepatic glutathione, which is necessary for the metabolism of NAPQI into non-toxic metabolites [2]. 

Fomepizole has occasionally been used in conjunction with NAC and acts by preventing the metabolism of APAP to NAPQI by inhibiting cytochrome p450 [4]. Treatment with fomepizole reduces the production of NAPQI by ~ 10-fold in healthy subjects [5]. In addition to limiting the production of NAPQI, fomepizole may prevent APAP-induced hepatotoxicity even after APAP is metabolized into NAPQI by inhibiting c-Jun N-terminal kinase (JNK), an enzyme that plays an important role in amplification of mitochondrial oxidant stress in APAP hepatotoxicity [6]. Fomepizole has minimal side effects, and the theoretical benefit for preventing hepatotoxicity and excellent safety profile has led to increasing use of fomepizole as an adjunctive treatment for APAP toxicity [7, 8, 9]. 

APAP is highly dialyzable, and conventional intermittent hemodialysis has been utilized in conjunction with NAC and fomepizole for the management of massive APAP toxicity. Prior cases have demonstrated success in preventing APAP-induced hepatotoxicity using intermittent hemodialysis [7, 8, 9]. Few cases have utilized continuous veno-venous hemofiltration (CVVH) for APAP ingestion. Both NAC and fomepizole are highly dialyzable medications, and the ideal dosages of NAC and fomepizole while undergoing CVVH has not been well described in the literature. Therefore, recommendations are needed on the effective dosages of these drugs for managing tylenol ingestion in patients undergoing CVVH. 

We present a case of massive APAP ingestion treated with NAC, fomepizole, and hemodialysis followed by CVVH with adjusted rates and doses of NAC and fomepizole that resulted in a favorable outcome without the development of acute liver injury. 

## Case report 

**Figure 1.**
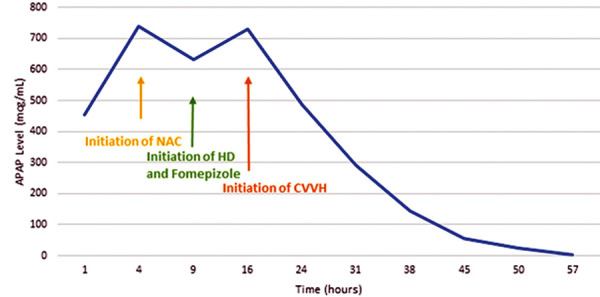
Acetaminophen levels over the course of time.

An 18-year-old male with a past medical history of major depressive disorder, generalized anxiety disorder, and borderline personality disorder presented 1 hour after consuming 125 grams of APAP. He arrived with symptoms of nausea and vomiting with a Glasgow coma score of 15. Initial labs were notable for an APAP level of 455.4 µg/mL, AST 18 U/L, ALT < 10 U/L, alkaline phosphatase 83 U/L, total bilirubin 1.4 mg/dL, INR 1.33, and ammonia 14 mcmol/L. Four hours after ingestion, his APAP level had risen to 738.6 µg/mL, and he was given 150 mg/kg of intravenous NAC followed by continuous intravenous NAC at 12.5 mg/kg/h. One hour later (~ 5 hours after ingestion), he developed shock (BP 76/35) and an acute decline in his mental status with a Glasgow coma score of 6. He was subsequently intubated for airway protection and started on two pressors (norepinephrine and vasopressin). Venous blood gas at this time was notable for a pH 7.27, pCO_2_ 37 mmol/L, and bicarbonate was 15 mmol/L. The toxicology consultant recommended treatment with fomepizole and hemodialysis given the patient’s rapid deterioration. The APAP level prior to initiating hemodialysis and fomepizole was 630.6 µg/mL. After completing a 4-hour run of hemodialysis and a one-time 15-mg/kg dose of intravenous fomepizole, his APAP level rose to 730 µg/mL. The patient was subsequently started on CVVH 4 hours later due to his transfer to a neighboring hospital. Eight hours after initiating CVVH at a high replacement fluid rate of 90 mL/kg/h, his APAP level dropped to 487 µg/mL. Due to the high fluid replacement rate for CVVH, the dosing of NAC and fomepizole were adjusted. In addition to intravenous NAC at 12.5 mg/kg/h, the patient was started on oral NAC at 70 mg/kg every 4 hours, and the frequency of intravenous fomepizole dosing was increased to 10 mg/kg every 6 hours. APAP levels were routinely monitored until an undetectable level (Figure 1). His INR, AST, and alkaline phosphatase peaked 2 days after ingestion at 2.25, 50 U/L, and 76 U/L, respectively. His ALT peaked 4 days after ingestion at 55 U/L. NAC, CVVH, and fomepizole were discontinued 3 days after ingestion once his APAP levels became undetectable. He was subsequently extubated and returned to his baseline mental status. Liver enzymes returned to normal. 

## Discussion 

APAP toxicity can be fatal if acute liver failure develops. Fortunately, this can be prevented with appropriate treatment that is given in a timely manner. Extensive literature demonstrates the value of early treatment with NAC in APAP poisoning. A growing body of animal studies, healthy subject studies, and case reports/series supports the addition of fomepizole in cases where NAC alone may be insufficient for preventing hepatotoxicity, such as patients with massive APAP ingestions or late-presenting patients. The early treatment with NAC and the addition of fomepizole likely protected this patient from APAP-induced hepatic injury. Although not routine practice, the addition of dialysis to NAC and fomepizole has shown promising outcomes in preventing liver toxicity from APAP ingestion. Additionally, intermittent hemodialysis and CVVH have been utilized in massive tylenol ingestion in which patients become acidotic. Several of the previous cases treated with NAC, fomepizole, and dialysis were managed with a single intermittent hemodialysis session. Many of these cases had a significant reduction in APAP levels with some even becoming undetectable after a single session [7, 8, 9]. 

The EXTRIP workgroup guidelines for APAP poisoning suggest that extracorporeal therapy be performed for patients with metabolic acidosis and APAP concentration greater than 900 µg/L in patients receiving NAC therapy. However, given the precipitous deterioration in clinical status and the fact that the APAP concentration increased from 630.6 to 730 µg/mL despite 4 hours of intermittent hemodialysis, we opted to pursue extracorporeal treatment as the patient’s APAP level was likely going to exceed 900 mg/L. The rise in APAP level despite hemodialysis was likely due to continued absorption of acetaminophen from the GI tract and high tissue distribution of APAP in intestinal organs after oral ingestion [10]. Charcoal can be an additional therapy used to bind APAP and reduce its concentration. Charcoal was not administered to this patient upon arrival because of his rapidly deteriorating mental status, and the risk of aspiration was assessed to be too high. It took several hours to initiate dialysis and transfer to a neighboring hospital, and it was believed that the benefit of giving charcoal, after these tasks were accomplished, was minimal. Therefore, charcoal was not administered in this case. It is important to note, however, that charcoal can be used as an adjunctive therapy in APAP ingestion if provided early. 

CVVH was chosen in this case due to its ability to effectively remove mid-sized molecules such as that of protein-bound acetaminophen [11, 12]. Prior cases that implemented CVVH for the management of tylenol ingestion utilized different rates of NAC and fomepizole administration. In one report, NAC was not utilized as the patient presented 10 hours after ingestion. They did not provide continuous doses of fomepizole during CVVH. The AST and ALT in this case peaked in the thousands. The patient presented outside of the effective time window for the benefits of NAC, which likely contributed to the elevated liver enzymes, however; the lack of additional doses of fomepizole during CVVH was also likely responsible for the elevated liver enzymes [9]. Another case provided an intravenous NAC regimen of 12 mg/kg/h and fomepizole of 600 mg every 6 hours while on CRRT, which was similar to our case. However, this case had an unfavorable outcome likely because the patient present 16 hours after tylenol ingestion [13]. 

Given the outcomes of these previous cases, we selected an aggressive replacement fluid rate of 90 mL/kg/h matched with blood flow rate of 350 mL/min to achieve a high clearance rate. This comes with the unintended effect of high clearance of fomepizole and NAC. As a result, we elected to provide a continuous NAC infusion at 12.5 mg/kg/h and oral NAC of 70 mg/kg every 4 hours with intravenous fomepizole at 10 mg/kg every 6 hours while on CVVH. With these adjustments, the AST and ALT remained less than 100 U/L during the entire hospitalization, and no liver failure developed. CVVH, NAC, and fomepizole were discontinued when the APAP concentration was undetectable. We recommend these dose adjustments for NAC and fomepizole when managing patients for tylenol ingestion who are receiving CVVH as it has been proven to be effective. Notably, the benefits of NAC, fomepizole, and dialysis are limited by time as seen by previous cases that were not effective [13]. 

This case encourages the utilization of NAC, fomepizole, and CVVH in patients who experience a massive APAP ingestion. Based on this case, it can be proposed that CVVH with high-flow rates may be helpful in cases where intermittent hemodialysis is not effective or feasible for hemodynamic or logistical reasons. Fomepizole and NAC doses need to be adjusted as proposed in this case during CVVH when higher flow rates are used, as both antidotes are dialyzable. 

## Funding 

None. 

## Conflict of interest 

There is no conflict of interest to disclose.
